# Modulation of the skin microbiome in cutaneous T-cell lymphoma delays tumour growth and increases survival in the murine EL4 model

**DOI:** 10.3389/fimmu.2024.1255859

**Published:** 2024-04-05

**Authors:** Saptaswa Dey, Pablo Augusto Vieyra-Garcia, Aaroh Anand Joshi, Slave Trajanoski, Peter Wolf

**Affiliations:** ^1^ Department of Dermatology and Venereology, Medical University of Graz, Graz, Austria; ^2^ Core Facility Computational Bioanalytics, Medical University of Graz, Graz, Austria; ^3^ BioTechMed Graz, Graz, Austria

**Keywords:** CTCL (cutaneous T-cell lymphoma), skin microbiome, PUVA (combination of psoralen and long-wave ultraviolet radiation), UVB 311 nm, topical (local) antibiotics, adjuvant therapy, *Staphylococcus aureus*

## Abstract

Cutaneous T-cell lymphomas (CTCL) are a group of lymphoproliferative disorders of skin-homing T cells causing chronic inflammation. These disorders cause impairment of the immune environment, which leads to severe infections and/or sepsis due to dysbiosis. In this study, we elucidated the host-microbial interaction in CTCL that occurs during the phototherapeutic treatment regime and determined whether modulation of the skin microbiota could beneficially affect the course of CTCL. EL4 T-cell lymphoma cells were intradermally grafted on the back of C57BL/6 mice. Animals were treated with conventional therapeutics such as psoralen + UVA (PUVA) or UVB in the presence or absence of topical antibiotic treatment (neomycin, bacitracin, and polymyxin B sulphate) as an adjuvant. Microbial colonisation of the skin was assessed to correlate with disease severity and tumour growth. Triple antibiotic treatment significantly delayed tumour occurrence (*p* = 0.026), which prolonged the survival of the mice (*p *= 0.033). Allocation to phototherapeutic agents PUVA, UVB, or none of these, along with antibiotic intervention, reduced the tumour growth significantly (*p* = 0.0327, *p *≤ 0.0001, *p *≤ 0.0001 respectively). The beta diversity indices calculated using the Bray−Curtis model showed that the microbial population significantly differed after antibiotic treatment (*p *= 0.001). Upon modulating the skin microbiome by antibiotic treatment, we saw an increase in commensal *Clostridium* species, e.g., *Lachnospiraceae* sp. (*p *= 0.0008), *Ruminococcaceae* sp. (*p *= 0.0001)., *Blautia* sp. (*p *= 0.007) and a significant reduction in facultative pathogens *Corynebacterium* sp. (*p *= 0.0009), *Pelomonas* sp. (*p *= 0.0306), *Streptococcus* sp. (*p *≥ 0.0001), *Pseudomonas* sp. (*p *= 0.0358), and *Cutibacterium* sp. (*p *= 0.0237). Intriguingly, we observed a significant decrease in *Staphylococcus aureus* frequency (*p *= 0.0001) but an increase in the overall detection frequency of the *Staphylococcus* genus, indicating that antibiotic treatment helped regain the microbial balance and increased the number of non-pathogenic *Staphylococcus* populations. These study findings show that modulating microbiota by topical antibiotic treatment helps to restore microbial balance by diminishing the numbers of pathogenic microbes, which, in turn, reduces chronic inflammation, delays tumour growth, and increases survival rates in our CTCL model. These findings support the rationale to modulate the microbial milieu during the disease course of CTCL and indicate its therapeutic potential.

## Introduction

Cutaneous T-cell lymphoma (CTCL) represents a heterogeneous group of non-Hodgkin lymphomas characterised by the infiltration and expansion of neoplastic mature T cells, primarily in the skin (1). In these lymphoproliferative disorders, an impaired immune system is primarily responsible for more recurrent infections, chronic inflammation, and the suppression of antitumor activity (2, 3). External factors have been proposed as one of the key reasons for the aggravation of the disease (3–5). A recent hypothesis proposes that microbial antigens play a role in promoting chronic inflammation and malignant cell transformation (6); a similar role has been described in several other skin diseases, such as atopic dermatitis, psoriasis, and acne vulgaris (7–10). Advances in modern technologies, and especially in the sequencing methods such as 16s sequencing and whole-genome shotgun sequencing (11), equip researchers with more tools they can use to understand changes in skin-microbial populations up to the species level in different CTCL disease stages and during therapeutic interventions.

Intact human skin provides an effective barrier against environmental effects. The superficial layer of human skin is colonised by a plethora of microorganisms, comprising bacteria, archaea, fungi, and viruses, which form a mutualistic symbiosis. The balance of this heterogenous microbial community is essential for protecting the organism against invading pathogens and the breakdown of natural products (12). The microbiota can modulate the production of various anti-microbial peptides (AMPs), cytokines, and chemokines in the skin (5, 9, 12, 13). Commensal skin microbiota, including bacteria such as *Staphylococcus hominis* (SH) or *S. epidermidis* (SE) (14), ubiquitously colonise human skin and are non-harmful to humans. In contrast, *Staphylococcus aureus* (SA) is associated with various pathogenic skin conditions in humans. SA-derived enterotoxins have evoked particular interest because they belong to a class of “superantigens”, which are exceptionally potent activators of T cells. If the skin barrier is breached or the immune system is impaired due to the expansion of malignant T cells in the skin (as in the case of CTCL), this delicate balance between commensal and pathogenic microorganisms is disrupted; this, in turn, triggers chronic inflammation which aggravates the disease phenotype. Furthermore, a chronic pro-inflammatory micro-environment has been shown to promote malignant T cell proliferation (2, 6, 15).

Most patients with early-stage mycosis fungoides (MF), the most common form of CTCL, characteristically present with cutaneous patches and plaques. These patients are treated with phototherapy to clear skin lesions and increase their disease-free survival rates. Narrowband UVB (NB-UVB) and psoralen plus UVA (PUVA) are the two primary forms of phototherapy used to treat these CTCL patients (16–21). Regarding leukemic CTCL (L-CTCL), extracorporeal photopheresis (ECP) is usually prescribed (21, 22) as therapy. UV radiation has been experimentally proven to have profound qualitative and quantitative influences on the composition of the skin microbiome (23, 24). Moreover, a study from our lab showed that the skin microbiome regulates the effect of UV radiation on cellular response and immune function (25). For these reasons, understanding the skin microbiome and host-microbial interactions during the phototherapeutic treatment regime in CTCL is extremely important. For instance, we have recently shown that PUVA induces local type 1 interferon production and antitumor response in CTCL patients, and intriguingly, rescued deficient interferon production may help in fighting infection with *Staphylococcus aureus* (SA), a driving factor in the pathophysiology of the disease (26).

The dysbiosis observed in the microbiome of CTCL patients is considered more than mere coincidence. Studies suggest that alterations in the microbiome can influence immune dysregulation, inflammation, and the progression of CTCL (4, 9, 15). Furthermore, microbial metabolites have been found to modulate T-cell responses and affect tumour microenvironments, potentially impacting disease outcomes (27, 28). The cause of malignant cell transformation in CTCL remains to be elucidated, but multiple factors are associated with the disease progression, such as chromosomal aberrations, oncogenic mutations, environmental factors, and the microbiome. Increased STAT3/5 signalling, resulting from copy number gains on chromosome 17q, and the loss of negative regulators along the JAK/STAT pathway, such as suppressors of STAT1 and SOCS1, are possibly key genetic factors that contribute to increased clonal expansion in leukemic cutaneous T-cell lymphoma. Due to these genetic abnormalities in the T cell, the immune defence system becomes severely impaired, and patients with advanced forms of the disease often die because of infection rather than lymphoma. Previous studies have demonstrated the crucial role of the microbiome in modulating disease activity in CTCL. Notably, Fanok et al. (29) showed in a lymphoma mouse model comparing germ-free vs. SPF animals that the presence of microbiota can significantly accelerate disease progression.

Their findings indicate that CTCL development is markedly slower under sterile conditions, highlighting the detrimental impact of bacterial presence on disease severity. This pivotal study underscores the interaction between the microbiome and immune response in CTCL, aligning with our current investigation. Our research, employing a distinct model and interventions, further explores this link, providing additional evidence that modulating the skin microbiome through antibiotics and phototherapy can delay tumor growth and improve survival outcomes. These independent yet complementary approaches reinforce the concept that targeting microbial influences offers a promising avenue for therapeutic intervention in CTCL (29). Intriguingly, severe bacterial infections are often seen in CTCL patients because malignant T cells also may induce significant changes in the skin architecture; this, in turn, impairs the skin barrier function, increasing the patient’s susceptibility to bacterial infections and their spread. Recent research, has provided insight into this process, showing how primary malignant T cells induce significant changes in the expression of skin barrier proteins in CTCL through cytokine-mediated JAK/STAT signaling, highlighting the intricate relationship between malignant T cell activity and compromised skin barrier integrity in the disease pathology of CTCL (30). SA has also been presumed to play a tumor-promoting role since antibiotic treatment specific to SA has been shown to have an inhibitory effect on the tumor burden in some patients. This observation aligns with the findings by Gluud et al. (31), further supporting the notion of targeted antibiotic therapy as a viable approach to modulate disease progression in CTCL (32).

This study was carried out to elucidate the host-microbial interaction occurring during the phototherapeutic treatment of CTCL, to support the rationale for modulating the microbial milieu during the disease course of CTCL, and to indicate the therapeutic potential of such modulation.

## Results

### Modulation of the skin microbiome with topical triple antibiotic treatment delays tumour occurrence and increases survival rates in a cutaneous T-cell lymphoma mouse model

**Figure 1:**
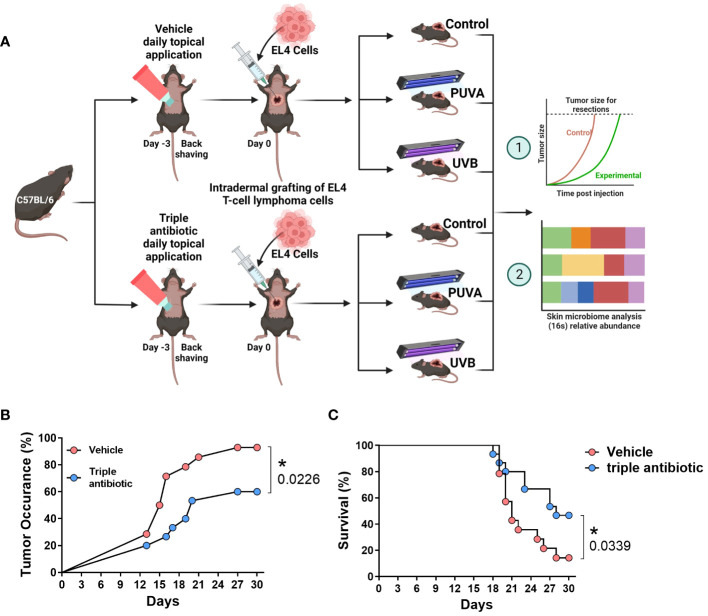
Modulation of the skin microbiome with topical triple antibiotic intervention delays the tumour occurrence and increases the survival rate in the cutaneous T-cell lymphoma mouse model. **(A)** Graphical schematic of the mouse experimentation model. **(B)** Tumour occurrence (%) upon topical triple antibiotic intervention as compared with the vehicle-treated control group (*n* = 15). **(C)** Kaplan–Meier survival analysis results comparing the effect of topical triple antibiotic intervention in the antibiotic-treated group and the vehicle-treated control group (*n* = 15). Survival rates were calculated based on the day mice were sacrificed. Individual mice were sacrificed once the tumour reached 10 mm in diameter, as defined in the study protocol.

**Figure 2:**
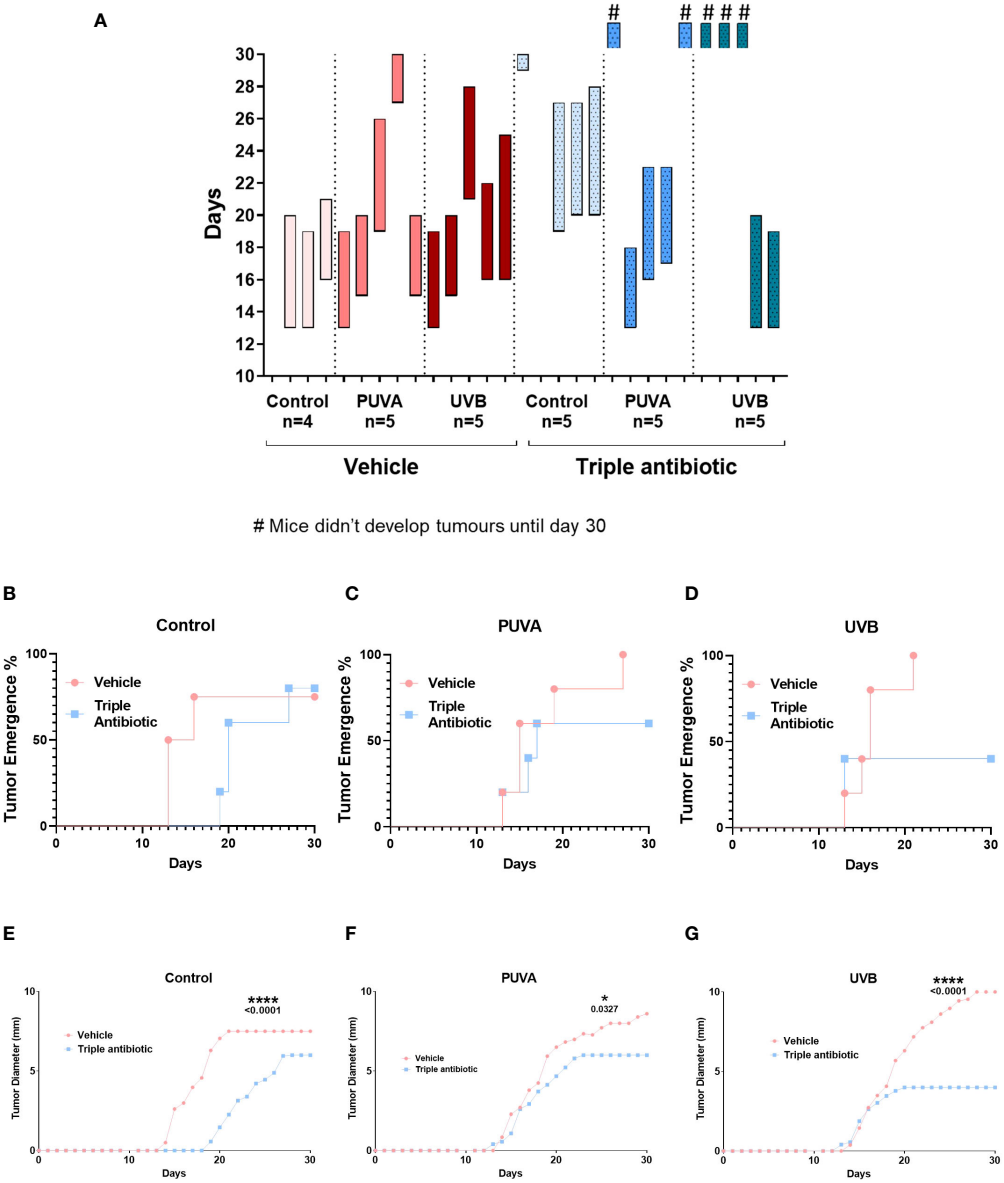
Phototherapy and topical triple antibiotic intervention as an adjuvant significantly reduced the tumour occurrence, diameter, and growth rate. **(A)** Tumour timeline: Bars indicate the time from tumour implementation until the death of individual mice, comparing to control, PUVA, and narrowband UVB therapy subgroups in the presence or absence of microbial modulation by triple antibiotic application (*n* = 4–5 per experimental group). Bars with symbol # indicates the mice which didn’t develop visible tumours and were terminated due to end of experiment. Tumour emergence (%) plot of vehicle- vs triple antibiotic-treated mice **(B)** control, **(C)** PUVA, and **(D)** UVB (*n* = 5 per experimental group). Tumour growth curve plotted as tumour diameter in (mm) for the vehicle- vs triple antibiotic-treated group for **(E)** control, **(F)** PUVA, and **(G)** UVB (n = 5 per experimental group). Statistical significance was calculated using two-way ANOVA.

We intradermally grafted murine EL4 T-cell lymphoma cells onto the backs of the C57BL/6 mice and treated them with psoralen plus UVA (PUVA) or UVB in the presence or absence of topical triple antibiotic treatment. We then measured the microbial populations and correlated these with the disease severity and tumour growth (**Figure 1A**). Microbial modulation by topical triple antibiotic treatment reduced the tumour occurrence from 93% in the vehicle group to 60% in the antibiotic-treated group (*p* = 0.0226) (**Figure 1B**). Results of a Kaplan–Meier survival analysis of the murine CTCL model showed a significant increase in survival rates, namely 14.3% in the vehicle group as compared to 46.6% in the topical antibiotic-treated group (*p* = 0.0339) (**Figure 1C**). Adjuvant therapy of antibiotic intervention along with PUVA, UVB, or neither of these showed reduced tumour occurrence and delayed tumour growth (**Figure 2A**). The tumour emergence rates in the CTRL, PUVA, and UVB groups were 80%, 60%, and 40%, respectively, in antibiotic-treated mice as compared to 75%, 100%, and 100% in vehicle-treated mice (**Figures 2B–D**). Tumours were also fast-growing in vehicle-treated mice as compared to mice receiving antibiotic intervention (**Figure 2A**). A comparison of the tumour growth curve plotted as tumour diameter in (mm) for the vehicle- vs triple antibiotic-treated group with the control (**Figure 2E**), PUVA (**Figure 2F**), and UVB (**Figure 2G**) groups showed a remarkably significant reduction in the tumour diameter in all three groups (CTRL, p ≤ 0.0001; PUVA, *p* = 0.0327; UVB, *p* ≤ 0.0001). Furthermore, the results of the AUC (Area Under the tumour growth Curve) analysis show that antibiotic application reduced the growth rate of the tumour in all three groups (i.e. Control, PUVA, and UVB) (**Supplementary Figures 1A–C**). Finally, the antibiotic intervention increased survival rates regardless of the phototherapeutic regime, as shown by the Kaplan-Meier survival analysis results (**Supplementary Figures 2A–C**) for the control (25%), PUVA (20%), and UVB (0%) subgroups within the vehicle-treated group and the control (40%), PUVA (40%), and UVB (60%) subgroups within the antibiotic-treated group.

### Antibiotic intervention of CTCL tumours alters the skin microbial diversity and richness irrespective of the phototherapeutic regime

**Figure 3:**
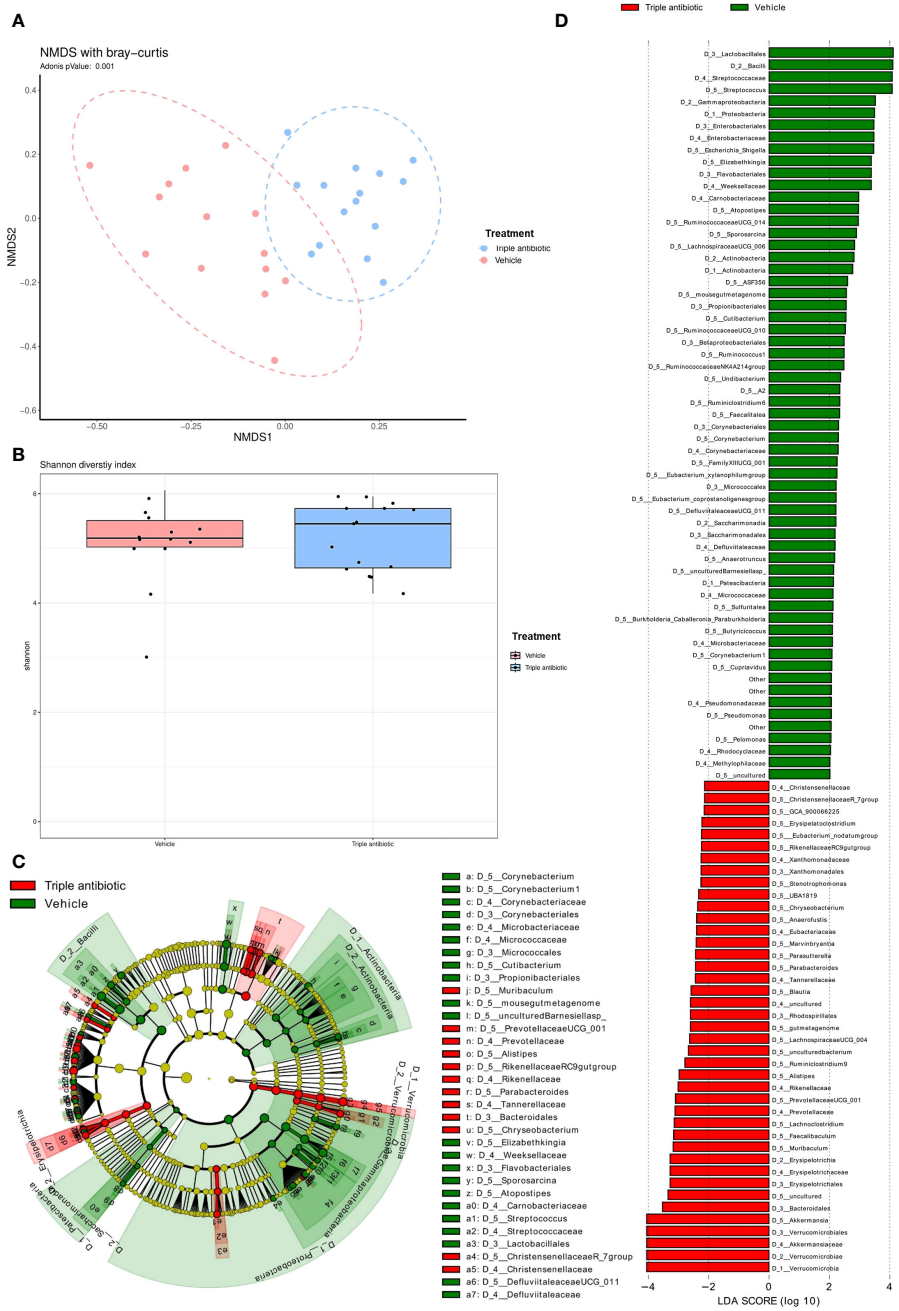
Topical triple antibiotic application on the CTCL tumour alters the microbial population on the skin, resulting in a significant increase in skin commensal communities and a reduction in the facultative pathogens. **(A)** Beta diversity analysis results obtained by performing non-metric multidimensional scaling (NMDS) with the Bray−Curtis model and 16S microbial sequencing data show the difference between the groups in the presence or absence of antibiotic intervention. (*n* = 15 per group). **(B)** Comparison of microbial diversity indices (i.e., Shannon diversity index) between the control vs antibiotic-treated group (*n* = 15 per group). **(C, D)** Linear discriminant analysis Effect Size (LefSe) analysis results: **(C)** Cladogram shows the taxonomic representation of statistically and biologically consistent differences between the control (green) and antibiotic-treated (red) groups (*n* = 15 per group). **(D)** Histogram of the Linear Discriminant Analysis (LDA) scores computed for differentially abundant features (LDA score [log_10_] 2) between the control (green) and antibiotic-treated (red) groups (*n* = 15 per group).

We analysed the microbial population in the lesional skin of our CTCL model by performing 16s microbial sequencing. Results of a beta diversity analysis performed by non-metric multidimensional scaling (NMDS) with the Bray-Curtis model show a significant difference in microbial population upon antibiotic treatment compared to the vehicle-treated group (*p*=0.001) (**Figure 3A**). A comparison of the microbial diversity indices (i.e., Shannon diversity index) shows that microbial diversity was slightly increased in the antibiotic-treated group as compared to the vehicle-treated group; this higher diversity and evenness indicated healthier skin (**Figure 3B**). We performed a Linear discriminant analysis Effect Size (LefSe) analysis which shows a higher number of facultative pathogens, e.g. *Pelomonas* sp. (*p* = 0.0306) *Corynebacterium* sp. (*p* = 0.0009) (**Supplementary Data 1**). These species have been reported to be associated with pain signatures in CTCL lesional skin (33) (**Figures 3C, D**). The cladogram shows a taxonomic representation of statistically and biologically consistent differences between the vehicle- and antibiotic-treated groups (**Figure 3C**).

**Figure 4:**
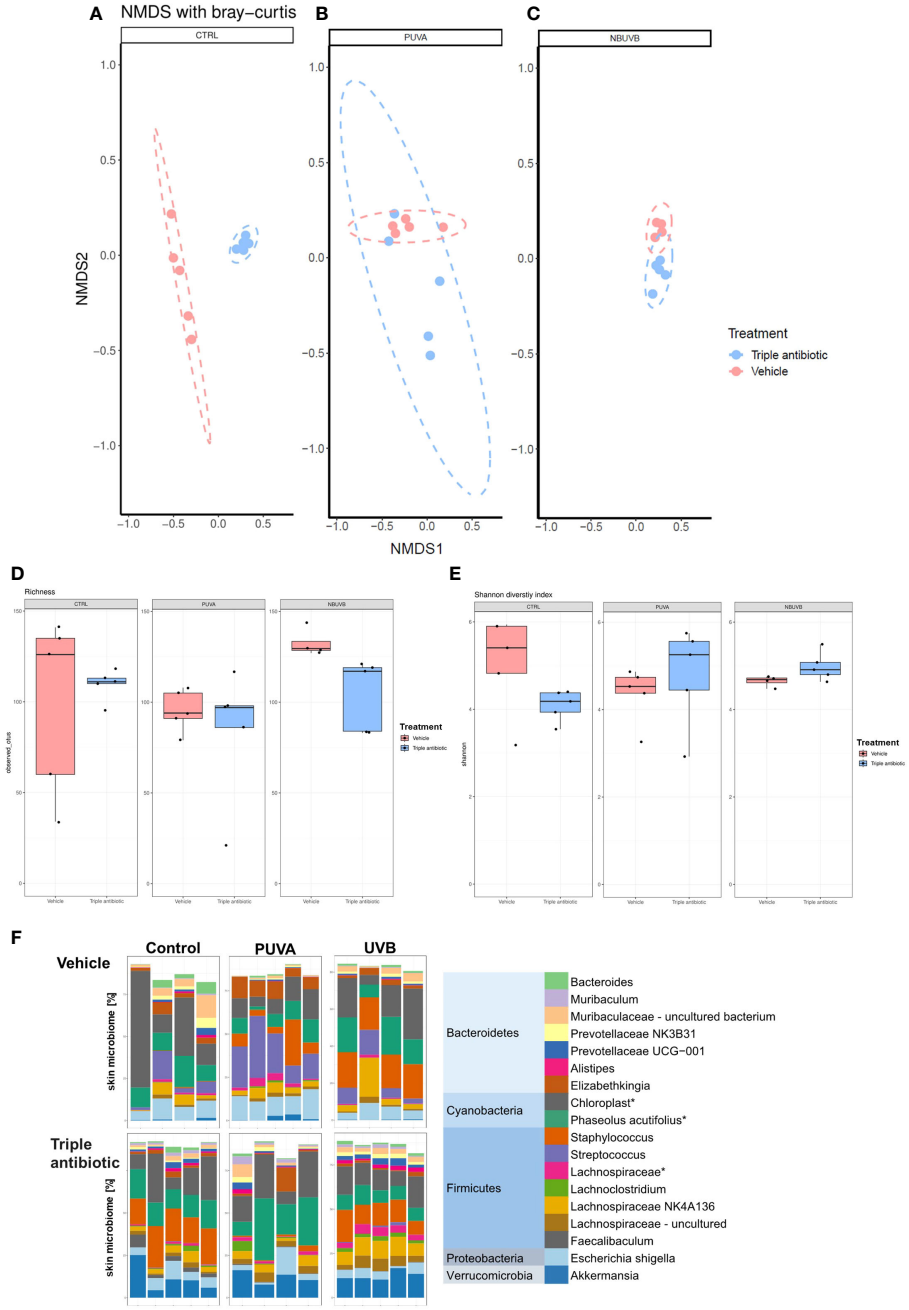
Antibiotic intervention of CTCL tumours alters the microbial diversity and richness regardless of the therapeutic regime used. The results of a non-metric multidimensional scaling (NMDS) analysis of 16S microbial sequencing data show the difference between different treatment groups **(A)** CTRL, **(B)** PUVA, and **(C)** UVB) in the presence or absence of antibiotic intervention as adjuvant therapy (*n* = 5 per experimental group). Box plot showing the differences in terms of **(D)** microbial richness (observed OTUs) and **(E)** microbial diversity indices (i.e., Shannon diversity index) regarding the vehicle-treated and topical triple antibiotic intervention group in the phototherapeutic treatment subgroups (UVB/PUVA/CTRL) (*n* = 5 per experimental group). **(F)** Relative abundance of the 18 most abundant genera (histogram; different genera represented by different colours) revealed by an analysis of the 16S sequencing data, grouped by presence or absence of antibiotic intervention as adjuvant therapy in the phototherapeutic treatment regime (*n* = 5 per experimental group).

Results of a beta diversity analysis of (NMDS) 16S microbial sequencing data performed with the Bray−Curtis model show this difference and enable a comparison to be made between the CTRL groups with or without antibiotic intervention (**Figure 4A**). However, the reduction in beta diversity observed in the PUVA and UVB groups in the presence or absence of antibiotic intervention (**Figures 4B, C**) was not unexpected, as UV therapy is known to be bactericidal. Furthermore, the microbial richness analysis results show a reduction in microbial richness in all three groups (CTRL, PUVA, and UVB) upon antibiotic intervention (**Figure 4D**). An analysis of the Shannon diversity indices, however, interestingly shows an increase in the diversity of the antibiotic intervention group when combined with PUVA or UVB (**Figure 4E**) but in contrast there is decrease in antibiotic treated monotherapy group, which might be due to the low sample size (n=4) in this group. Because the Shannon diversity index is a calculation of the number of species in a community and provides a measure of the evenness, the improved Shannon indices seen in the PUVA and UVB groups indicate that, although the species richness is reduced, the species evenness improved when both phototherapy and antibiotic treatment were used. These findings indicate a lower probability that a single species will dominate the CTCL lesional skin when antibiotic intervention is used with a phototherapeutic treatment regimen. If we examine the relative abundance of the 18 most abundant genera (**Figure 4F**) (histogram; different genera represented by different colours) based on the 16S sequencing data, an overall decrease in the abundance of *Streptococcus* sp. and an increase in the abundance of *Staphylococcus* sp. is evident in the antibiotic intervention group.

**Figure 5:**
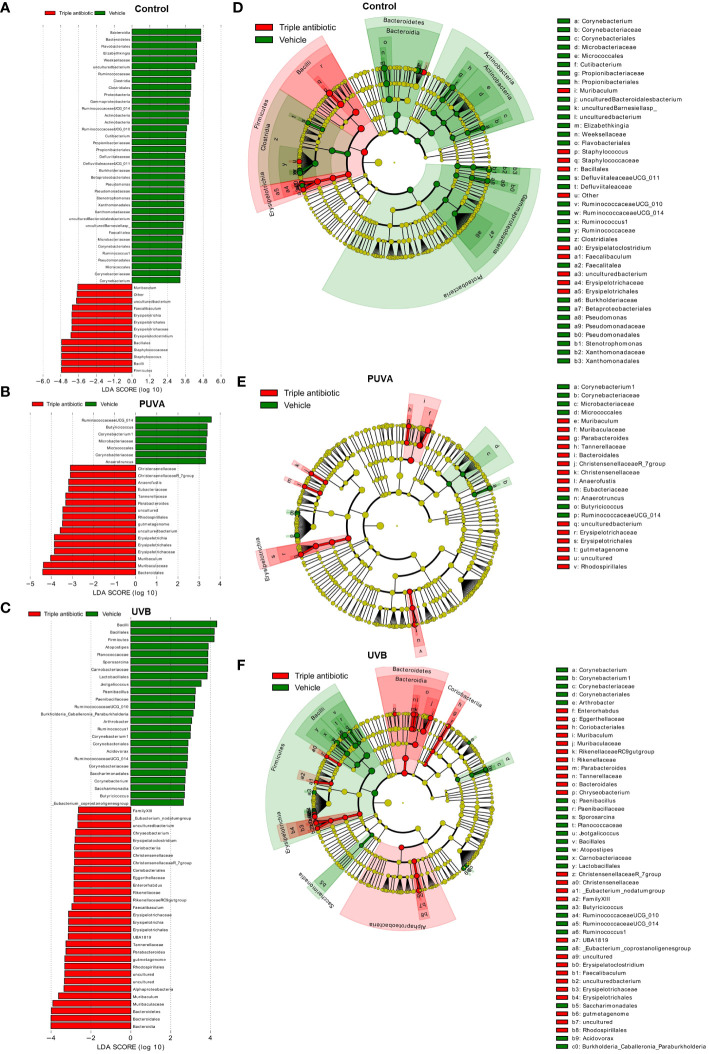
Significantly altered taxonomic differences among three phototherapeutic treatment groups (control, PUVA, and UVB) in the presence or absence of antibiotic intervention as an adjuvant. Linear discriminant analysis Effect Size (LefSe) analysis results: Histogram of the Linear Discriminant Analysis (LDA) scores computed for differentially abundant features (LDA score [log 10] 3) between the vehicle- (green) and triple antibiotic-treated (red) groups are plotted for **(A)** control, **(B)** PUVA, and **(C)** UVB (*n* = 5 per experimental group). The cladogram shows the differences in terms of enriched taxonomic representation between the control (green) and topical triple antibiotic-treated (red) groups in **(D)** control, **(E)** PUVA, and **(F)** UVB (*n* = 5 per experimental group).

The results of the LefSe analysis of data from the phototherapeutic treatment groups in the presence or absence of antibiotic intervention show a significant difference (LDA score [log 10] 3) in terms of their taxonomic features: *Corynebacterium* sp. is more highly abundant in the vehicle-treated groups of CTRL, PUVA, and UVB (**Figures 5A–C**) than the respective groups with antibiotic intervention. The cladogram shows the statistically and biologically consistent differences in the taxonomic representation among the control, PUVA, and UVB groups (**Figures 5D–F**).

### Topical triple antibiotic intervention on CTCL lesional skin alters the microbial population by significantly reducing facultative pathogens and increasing skin commensals

**Figure 6:**
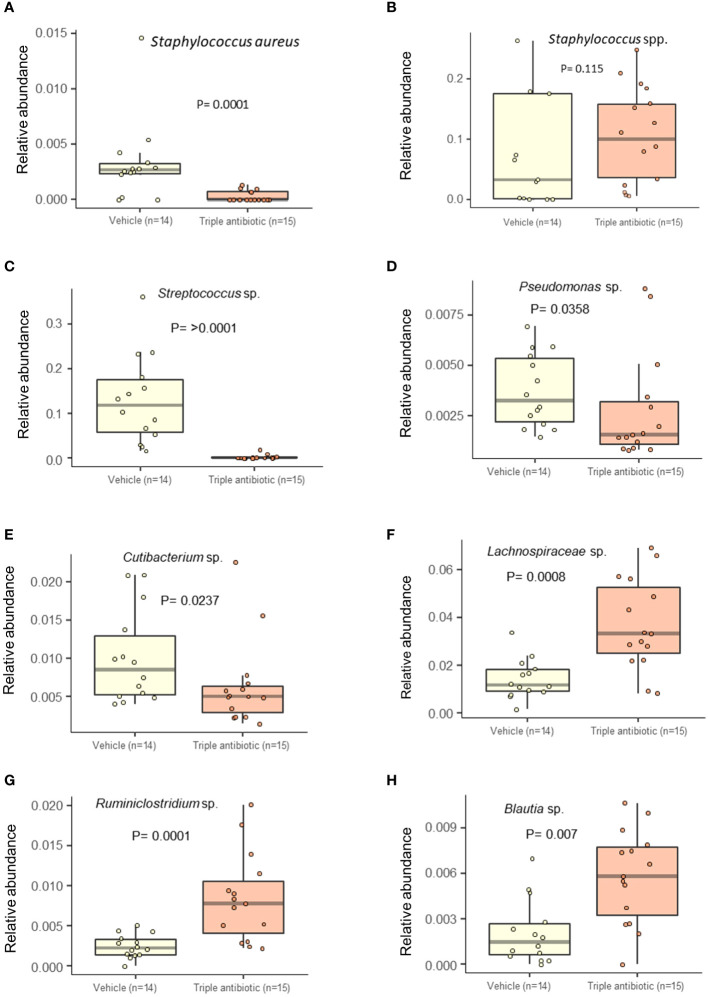
Antibiotic intervention significantly decreases the abundance of facultative pathogens and increases the abundance of commensal bacteria. Alteration in the relative abundance of the *Staphylococcus* spp. **(A)** *Staphylococcus aureus* **(B)** *Staphylococcus* genus. Reductions in the relative abundance of the following facultative pathogens were observed: **(C)** *Streptococcus* sp., **(D)** *Pseudomonas* sp., and **(E)** *Cutibacterium* sp. An increase in the abundance of the following commensal *Clostridium* species was observed: **(F)** *Lachnospiraceae* sp., **(G)** *Ruminococcaceae* sp., **(H)** *Blautia* sp. upon antibiotic treatment (*n* = 14–15 per experimental group).

A multivariate analysis performed by running a linear models (MaAsLin) analysis on 16s data revealed that antibiotic intervention reduced the detection frequency of *Staphylococcus aureus* in all three groups, i.e., CTRL, PUVA, and UVB (*p* = 0.0001) (**Figure 6A**). However, an interesting overall increase in the detection frequency of *Staphylococcus* genus upon antibiotic intervention was observed (*p* = 0.115) (**Figure 6B**), indicating that this intervention helped the mice to regain microbial balance by diminishing the frequency of pathogenic *Staphylococcus aureus* and increasing the frequency of likely non-pathogenic *Staphylococcus* species. Due to limited depth of our 16s sequencing we were unable to specifically indicate the non-pathogenic *Staphylococcus* species such as *S. hominins* or *S. epidermidis*. Furthermore, the results of a MaAsLin analysis show a significantly high abundance of facultative pathogens, including *Streptococcus* sp. (*p* ≥ 0.0001) (**Figure 6C**), *Pseudomonas* sp. (*p* = 0.0358) (**Figure 6D**), and *Cutibacterium* sp. (p = 0.00237) (**Figure 6E**) in the vehicle group compared to the antibiotic-treated group. In contrast, we saw a higher abundance of *Clostridium species*, e.g., *Lachnospiraceae* sp. *(p = 0.0008)* (**Figure 6F**) *and Ruminococcaceae* sp. *(p = 0.0001).* (**Figure 6G**)*, Blautia* sp. *(p = 0.007)* (**Figure 6H**) in the antibiotic-treated group, which is known to be T_reg_-inducing in inflammatory conditions (34). The abundance of several other microbial species belonging to the facultative skin microbiome, or the gut microbiome, decreased on mouse skin upon topical triple antibiotic application (e.g., *Elizabethkingia* sp., *Undibacterium* sp., *Serratia* sp., *Escherichia Shigella* sp., *Ruminococcus.* sp., and species in the families *Lachnospiraceae*, *Ruminococcaceae*, and *Methylophilaceae*) (**Supplementary Figures 3A–H**). Furthermore, the abundance of several microbial species increased significantly on mouse skin, indicating that microbial balance was regained upon tropical triple antibiotic application (e.g. *Alistipes* sp., *Akkermansia* sp., *Ruminiclostridium* sp.*, Muribaculum* sp., *Rhodospirillales* sp., *Parabacteroides* sp., *Faecalibaculum* sp., *Marvinbryantia* sp., *Lachnoclostridium* sp., *Parasutterella* sp., *Blautia* sp., and species in the families *Erysipelotrichaceae*, *Lachnospiraceae*, Ruminococcaceae, *Prevotellaceae*, and *Rikenellaceae*) (**Supplementary Figures 4A–N**), (**Supplementary Data 2**).

**Figure 7:**
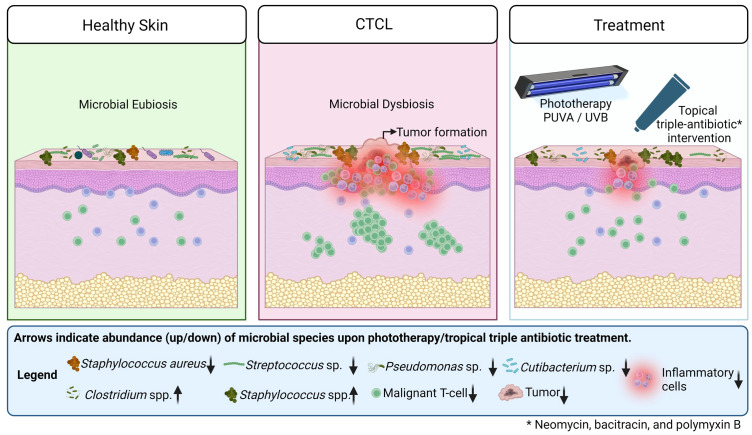
A graphical representation of the differences between healthy, CTCL lesional skin and CTCL lesional skin upon treatment. Arrows indicate abundance (up/down) of microbial species upon phototherapy along with topical antibiotic treatment as an adjuvant.

In summary, chronic inflammation, disruption of skin barrier function, and immune system impairment in CTCL skin lesions disrupt microbial eubiosis and promote the growth of facultative pathogens, e.g., *Staphylococcus* aureus, *Streptococcus* sp., *Pseudomonas* sp., *Cutibacterium* sp. Phototherapeutic treatment along with topical antibiotic intervention as an adjuvant, helps to restore microbial balance by reducing the number of pathogenic microbes and increasing the number of commensals, which in turn reduces chronic inflammation, delays tumour growth, and increases survival rate (**Figure 7**).

## Discussion

New evidence suggests that the skin microbiome in CTCL patients differs significantly from that of healthy individuals. Studies have reported decreased microbial diversity and alterations in the relative abundance of specific bacterial species in CTCL-affected skin (5, 9, 33). Notably, *Staphylococcus aureus* (SA) colonisation (6, 35) was observed in a subset of CTCL patients, exacerbating inflammation and contributing to disease progression. In CTCL, pronounced erythema in the lesional skin was associated with an increase in SA colonisation (6, 15, 35), and severe pain and lesion thickness were associated with the presence of *Corynebacterium* sp. and *Pelomonas* sp (33).

By using the EL4 T-cell lymphoma cutaneous mouse transplantation model, we could elucidate host-microbial interactions in CTCL during a phototherapeutic treatment regime and study the modulation of these interactions through antibiotic treatment. We observed that triple antibiotic treatment significantly delayed tumour occurrence and growth, which prolonged the survival of mice in the model, irrespective of the allocation to standard therapeutic agents (PUVA, UVB). An analysis of the beta diversity index obtained by applying the Bray−Curtis model showed that the microbial population significantly changed upon antibiotic treatment. This change was linked to an increase in the numbers of T_reg_-inducing commensal *Clostridium species* (12, 34) and a significant reduction in the numbers of the facultative pathogenic *Corynebacterium*, *Pelomonas*, *Streptococcus*, *Pseudomonas*, and *Cutibacterium* species. Interestingly, we observed a significant decrease in the detection frequency of *Staphylococcus aureus* but an increase in overall number of *Staphylococcus* genus, indicating that antibiotic treatment helped mice to regain microbial balance and increased the numbers of non-pathogenic *Staphylococcus* populations, which per se enables the mice to regain microbial eubiosis state (27).

Given the observed dysbiosis and the potential role of the microbiome in CTCL progression, the use of antibiotics may be proposed as a therapeutic approach. Antibiotics could modulate the microbiome, target specific bacterial species, or alter the microbial milieu to favour a more beneficial composition. The complex interplay between the immune system and the microbiome has been recognised by an increasing number of investigators as having a significant impact on human health and disease (35). In recent years, researchers have begun to shed light on the role of the microbiome in CTCL and the potential use of antibiotic intervention to modulate the microbiome in managing this disease (5, 31).

We have explored the impact of the skin microbiome modulation, through antibiotics and phototherapy, on disease progression in CTCL. In light of the groundbreaking findings by Vadivel et al. (36), demonstrating that *S. aureus* can induce drug resistance in malignant T cells, our study gains additional significance. This resistance is mediated through pathways previously implicated in CTCL pathogenesis and treatment resistance, notably TCR, NFκB, and JAK/STAT signaling. Given the established antibacterial effects of phototherapy, its role in CTCL treatment may extend beyond direct antineoplastic activity, potentially also mitigating bacterial-driven drug resistance. Vadivel et al.’s findings underscore the complex interplay between microbial pathogens, immune responses, and therapeutic resistance in CTCL. Therefore, our data suggest that combining phototherapy with targeted antimicrobial strategies could offer a twofold benefit: direct tumor cell cytotoxicity and disruption of bacterial-mediated resistance mechanisms. This approach, opens new avenues for enhancing the efficacy of existing and future therapeutic strategies against CTCL, warranting further investigation (36).

In another recent pivotal study by Liu et al. (37), the characteristics of *S. aureus* colonization in CTCL were extensively examined, revealing significant findings that enhance our current understanding of the microbiome’s impact on CTCL. The study included a substantial cohort of over 60 patients, providing a robust dataset for analysis. Liu et al. discovered that S. aureus colonization was present in a significant portion of CTCL patients and that the colonization rates increased with disease progression. Notably, the study also compared lesional and contralateral non-lesional skin sites, finding that *S. aureu*s colonization was more prevalent in lesional skin, which may suggest a potential role of bacterial presence in exacerbating disease severity (37). These findings align with our current study’s hypothesis on the microbiome’s influence on CTCL progression and highlight the importance of considering microbial factors in developing therapeutic strategies. Such insights are invaluable for informing future research and clinical practices, suggesting a potential benefit in targeting microbial colonization as part of comprehensive CTCL treatment plans.

The recent advancements in understanding the interplay between microbial interactions and immune responses have significantly enriched our perspective on CTCL management. The recent study in collaboration with our lab by Yu et al. (26) and the commentary by Goel and Rook (38), have illuminated the promising role of PUVA therapy in enhancing type I IFN responses, a pathway also implicated in the antimicrobial response against Staphylococcus aureus as demonstrated by research highlighting the bacterium’s activation of type I IFN signaling through both its Xr domain (39) and via TLR9 in dendritic cells (40). Furthermore, the study on gamma interferon’s role in bolstering human endothelial cells against S. aureus infection (41) underscores the critical nature of IFN signaling in mediating resistance to microbial infections. These insights not only underscore the therapeutic potential of targeting microbial interactions and immune pathways in CTCL but also suggest a broader applicability in enhancing antitumor immunity and patient outcomes through integrated approaches that consider the microbiome’s influence on disease progression and treatment response.

In a human study, the bacterial groups *Bacteroides*, *Escherichia/Shigella*, and *Streptococcus* were found to be most prevalent in disease conditions such as polycystic ovary syndrome (PCOS) and obesity (42). In contrast, *Akkermansia* and *Ruminococcaceae* decreased in PCOS and showed opposite results for body weight, sex hormones, and brain-intestinal peptides (42, 43). In our study, also we see a significant decrease in *Escherichia/Shigella* and an increase in the detection frequency of *Akkermansia* and *Ruminococcaceae* upon antibiotic intervention, indicating the role of these microorganisms in disease severity in our model.

The mechanisms underlying the potential efficacy of antibiotics in CTCL are multifaceted. Antibiotics may directly affect bacterial species associated with disease progression, reduce pro-inflammatory stimuli, modulate immune responses, and alter the tumour microenvironment (15, 31). Additionally, antibiotics might influence the production of microbial metabolites that impact T-cell function and immune surveillance (31). For this reason, further host-microbial interaction studies are essential to provide support for the use of specific antibiotic treatment to mitigate CTCL symptoms. Further research should also focus on developing antibiotics or anti-microbial agents with improved specificity and a reduced impact on the commensal microbiome. Targeting specific bacteria associated with CTCL progressions, such as SA, *Corynebacterium* sp., and *Pelomonas* sp. while preserving the beneficial microbiota could provide a more precise approach.

Assessing the long-term effects of antibiotic interventions in CTCL is crucial. Prospective studies are needed to evaluate the extent of microbiome modulation, potential microbiome recovery after antibiotic cessation, and the impact of this treatment on disease progression and overall patient outcomes. The use of precision medicine strategies, including microbial profiling, genomics, and metabolomics (44), can help to identify patients who are more likely to respond to antibiotic interventions. Understanding the individualised characteristics of the microbiome and its interactions with the host immune system could also improve treatment outcomes.

While evidence suggests the clinical benefits of such treatments, further research is needed to elucidate the optimal antibiotic regimens, potential side effects, and long-term implications of antibiotic use. Understanding the complex interplay between the microbiome, antibiotics, and CTCL will contribute to the development of personalised treatment strategies for this challenging disease. We believe that this research describes a rationale for using specific antibiotic interventions to modulate the microbial milieu during the disease course of CTCL and indicates the therapeutic potential of such modulation. In fact, using specific antibiotics may be more effective than eradicating the entire cutaneous microbiome by using other disinfection methods, such as antiseptic whirlpool baths (27), preserve the landscape of commensals and ultimately contribute to a balanced immune response by supporting the production and release of anti-microbial peptides (24, 28, 45, 46).

One limitation of our work is that we did not perform 16s microbiome analysis of healthy untreated mice from our animal housing for baseline control purposes. However, we can refer to existing literature to understand the typical microbial diversity in the skin of normal C57BL/6 mice. Indeed, the gut and skin microbiome of C57BL/6 mice have been extensively studied, revealing that they host a diverse milieu of microorganisms. However, it is important to note that even the ‘normal microbiota’ can vary based on several factors, including the environment, diet, and genetics of the mice (47–50). For example, it was found that significant differences in microbiome of C57BL/6 mice from different vendors, indicating variability even within the same strain (47, 48). Moreover, it was demonstrated the impact of environmental factors affects the skin microbiome and immune signatures in C57BL/6 mice (49–51). Furthermore, Naik et al. highlighted the dynamic interaction between commensal microbiota and the cutaneous immune system, which is relevant to our study’s context of cutaneous T-cell lymphoma (50). Another limitation of our study is that its design did not resemble a treatment schedule once CTCL is diagnosed since it was started before tumor cell inoculation. However, the rationale for initiating antibiotic therapy 3 days prior to the tumoral challenge in our study was twofold: to establish a homogeneous microbial environment at the outset of tumor development and to assess the prophylactic and therapeutic potential of microbiome modulation in CTCL.

The skin microbiota could be modulated in several ways, including antibiotics (31), UV-C lamps (52–54), specific bacterial therapy (14, 55), probiotics (56, 57), or endolysin (58). Our study serves as an indicator that there is an unmet need for modulation of the microbiota along with conventional therapeutic approaches to reduce disease severity and improve survival.

## Materials and methods

### *In vivo* intradermal CTCL mouse model

Animal work was done in accordance with institutional guidelines on animal welfare and with the approval of the Austrian Federal Ministry of Science, Research and Economy (BMBWF-66.010/0042-V/3b/2019). Four-week-old C57BL/6 mice (strain Ncrl) were obtained from the Charles River Laboratories (Freiburg, Germany). Mice were maintained under specific pathogen-free (SPF) conditions in individually ventilated cages at the Biomedical Research Facility (BMF) at the Medical University of Graz, Austria. Mice were kept on a 12/12 h light cycle and received standard food and water *ad libitum*. At six weeks of age, mice were shaved on their backs (day -3 of experimental procedures) and randomised into two groups (with or without topical triple antibiotic application), *n* = 15 per group. Under isoflurane inhalation anaesthesia (1–1.5% in O2, 0.5 L/min), we then intradermally grafted murine 6x10^3^ EL4 T-cell lymphoma cells in the back skin of the mice on day 0. The mice were then further randomised into three subgroups: CTRL (untreated), PUVA, and UVB (*n* = 5/subgroup).

### Antibiotic intervention

Antibiotic intervention by a topical triple antibiotic cream (Neosporin**^®^** (neomycin, bacitracin, and polymyxin B sulphate)) or Vaseline^®^ (petroleum jelly). For each application, a precise amount of 50 mg of pre-weighted antibiotic cream or vehicle was administered. The antibiotic cream or vehicle was directly applied to the tumor area and gently spread over the tumor area and adjacent shaved skin. Application was started post-shaving on day -3 and given daily until scarification occurred.

### Phototherapy

PUVA or NB-UVB therapy was given every second day at a dose of 1500 mJ/cm^2^ (PUVA) or 200 mJ/cm^2^ (NB-UVB), starting at day 1 using Waldmann UVA 236 equipment (Waldmann GmbH, Villingen-Schwenningen, Germany). In the case of PUVA, mice were painted on their backs with 200 microliter 8-methoxypsoralen (8-MOP) (Sigma-Aldrich, St. Louis, MO) in ethanol (at a concentration of 0.1 mg/ml), as previously described (59, 60). The mice were then kept for 15 min in individual compartments of standard cages to allow penetration of 8-MOP before UVA irradiation.

### Microbiome sampling procedure

Microbiome samples were collected from the skin with sterile swabs 20 days after the EL-4 cell injection. The swab used for sampling was first submerged in a sterile buffer solution (0.15 mol/L NaCl with 0.1% Tween 20) and consequently brushed 20 times in the crosswise direction over the sampled skin site (i.e., tumour and tumour-adjacent non-lesional sites in the murine model).

### DNA extraction, library preparation

Microbial DNA was obtained using the QIAamp DNA Microbiome Kit (Qiagen) according to the manufacturer’s instructions. The 16s libraries were constructed from the DNA extracted from swab samples using a Nextera XT library prep kit and 1.5 ng as starting material.

### 16s sequencing, bioinformatics, and statistical analyses

Amplicons were sequenced at the ZMF Core Facility Molecular Biology in Graz, Austria, using an Illumina MiSeq platform. The analysis was performed with the Quantitative Insights into Microbial Ecology QIIME 2 software (Version 2019.7) (61) next-generation microbiome bioinformatics platform integrated into a personal Galaxy server using the Medical University Graz MedBioNode HPC cluster. After initial quality control of the raw sequence data was performed with FastQC and MultiQC, initial data preprocessing was performed with the DADA2 pipeline (62), which included quality filtering and adapter trimming, denoising data, and removing chimeric artefacts. A QIIME2 Naive Bayes classifier trained with the 16S rRNA SILVA 132 database (63) was used to provide taxonomic annotation for representative sequences from the Amplicon Sequence Variants (ASVs) discovered by applying the DADA2 workflow. Alpha diversity indexes (e.g. richness indices and the Shannon and Faith’s phylogenetic diversity index), as well as beta diversity distances (e.g. weighted and unweighted UniFrac distance metrics, the Bray-Curtis dissimilarity index, and the Jaccard index), were also calculated with QIIME2, whereas all further statistical downstream analyses and plotting were performed in the R 4.2.2 program (R Core Team, 2022) for statistical computing and graphic illustration. To detect significantly abundant taxa, we used LefSe (Linear discriminant analysis Effect Size) (64) and MaAsLin2 (Microbiome Multivariable Association with Linear Models) (65) tools from the Huttenhower lab (Harvard T.H. Chan School of Public Health, Boston, MA).

### Statistical analysis

GraphPad Prism 8 and the R platform were used to perform the statistical analyses. The threshold for statistical significance was set at *p* < 0.05 unless otherwise specified. *p*-value: <0.05 (*), <0.01 (**), <0.001 (***), <0.0001 (****).

### Graphical license

The graphical abstract (**Figure 7**) and schematics of the mouse experimentation model were created using BioRender.com under the agreement numbers: YU25IH4YPH and SJ25IKLT21.

## Data availability statement

The datasets presented in this study can be found in online repositories. Sequence data were deposited in the European Nucleotide Archive (ENA; BioProject No. PRJEB64180).

## Ethics statement

The animal study was approved by Austrian Federal Ministry of Science, Research and Economy (approval no. BMBWF-66.010/0042-V/3b/2019). The study was conducted in accordance with the local legislation and institutional requirements.

## Author contributions

SD: Conceptualization, Data curation, Formal analysis, Funding acquisition, Investigation, Methodology, Software, Validation, Visualization, Writing – original draft, Writing – review & editing. PV-G: Conceptualization, Data curation, Formal analysis, Funding acquisition, Investigation, Methodology, Software, Visualization, Writing – review & editing. AJ: Writing – review & editing. ST: Formal analysis, Software, Writing – review & editing. PW: Conceptualization, Funding acquisition, Project administration, Resources, Supervision, Validation, Writing – review & editing.

## Supplementary material

Supplementary Figure 1 The topical triple antibiotic application reduces the AUC (Area Under the tumour growth Curve): The area under the tumour growth curve was calculated for individual mice and plotted. **(A)** CTRL (without phototherapy), **(B)** PUVA- or **(C)** UVB-treated group (*n* = 5 per group).

Supplementary Figure 2 Topical triple antibiotic intervention increases survival regardless of phototherapeutic regime: Kaplan-Meier survival analysis of **(A)** CTRL (without phototherapy), **(B)** PUVA, **(C)** UVB subgroups in the presence or absence of antibiotic intervention (*n* = 5).

Supplementary Figure 3 The abundance of several microbial species altered on mouse skin upon topical triple antibiotic application. **(A)** *Lachnospiraceae* sp., **(B)** *Elizabethkingia* sp., **(C)** *Ruminococcaceae* sp., **(D)** *Undibacterium* sp., **(E)** *Serratia* sp., **(F)** *Cupriavidus* sp., **(G)** *Shigella* sp., **(H)** *Ruminococcus* sp., **(I)** *Methylophilaceae* sp.

Supplementary Figure 4 Abundance of several commensal microbial species increased significantly on mouse skin upon tropical triple antibiotic application: **(A)** *Alistipes* sp., **(B)** *Akkermansia* sp., **(C)** *Lachnoclostridium* sp., **(D)** *Muribaculum* sp., **(E)** *Rhodospirillales* sp., **(F)** *Erysipelotrichaceae* sp., **(G)** *Parabacteroides* sp., **(H)** *Faecalibaculum* sp., **(I)** *Marvinbryantia* sp., **(J)** *Ruminococcaceae* sp., **(K)**. *Parasutterella* sp., **(L)** *Prevotellaceae* sp.,**(M)** *Erysipelatoclostridium* sp., **(N)** *Rikenellaceae* sp.

Supplementary Data Sheet 1 A data sheet showing the list of significantly altered bacterial abundance in the vehicle- vs the triple antibiotic-treated groups based on the results of the LefSE analysis. *P*-value cutoff > 0.05, LDA score [log_10_] 2 (*n* = 14-15 per group).

Supplementary Data Sheet 2 A data sheet showing the list of altered bacterial abundance in the vehicle- vs the triple antibiotic-treated groups according to the MaAsLin2 analysis results. *P*-value cutoff > 0.05 (*n* = 14-15 per group).
