# Supplementary figures and images for: Modulation of the skin microbiome in cutaneous T-cell lymphoma delays tumour growth and increases survival in the murine EL4 model
Source: Front Immunol. 2024 Apr 5;15:1255859. doi: 10.3389/fimmu.2024.1255859 (PMC11026597; doi:10.3389/fimmu.2024.1255859)

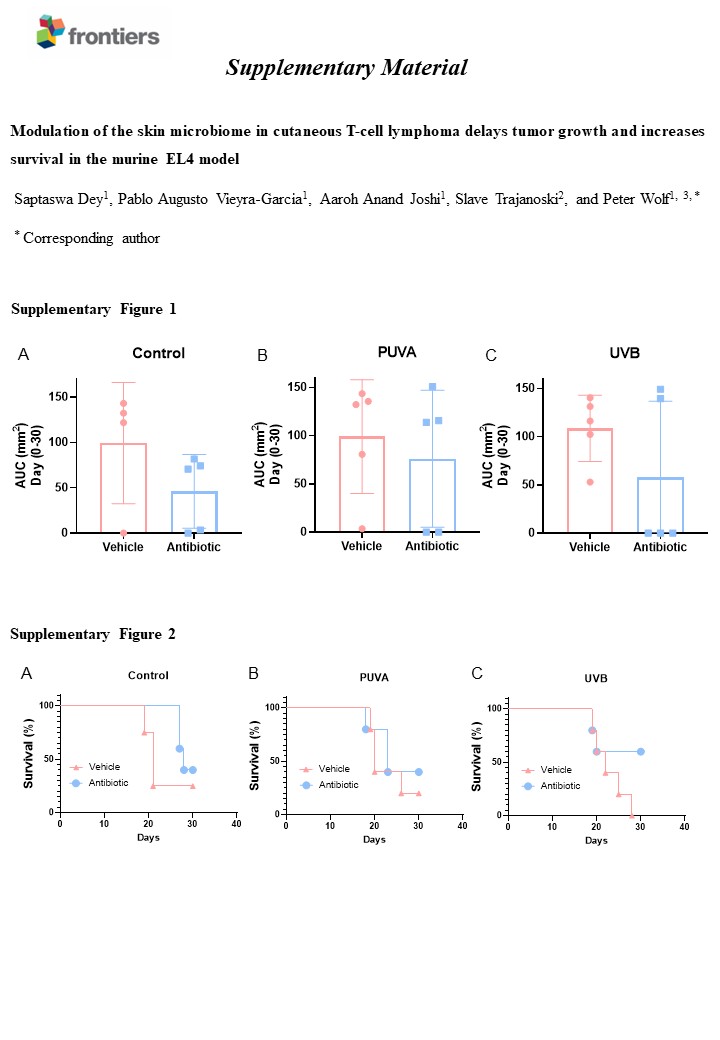

Supplement: Supplementary Figure 1 — The topical triple antibiotic application reduces the AUC (Area Under the tumour growth Curve): The area under the tumour growth curve was calculated for individual mice and plotted. (A) CTRL (without phototherapy), (B) PUVA- or (C) UVB-treated group (n = 5 per group). [file Image_1.jpg]

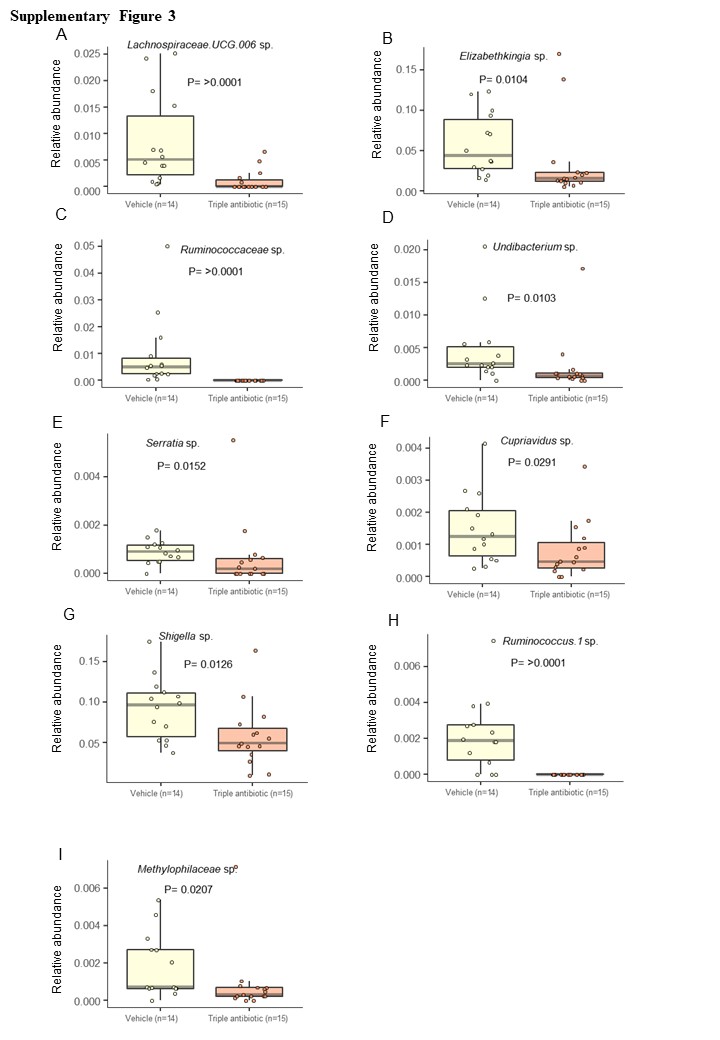

Supplement: Supplementary Figure 2 — Topical triple antibiotic intervention increases survival regardless of phototherapeutic regime: Kaplan-Meier survival analysis of (A) CTRL (without phototherapy), (B) PUVA, (C) UVB subgroups in the presence or absence of antibiotic intervention (n = 5). [file Image_2.jpg]

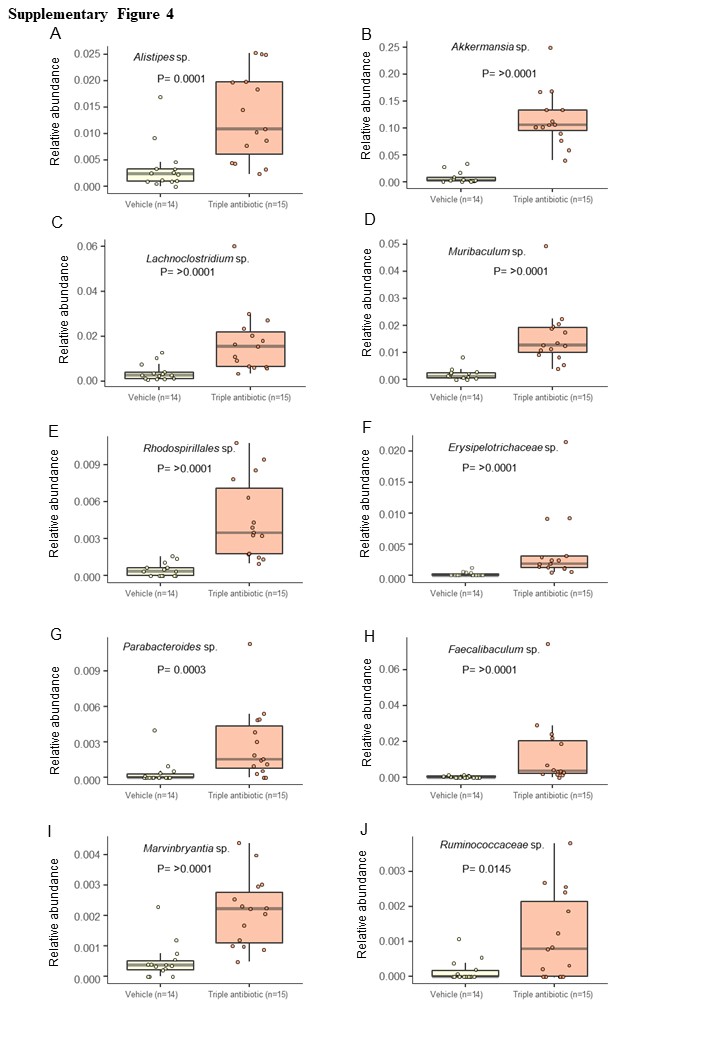

Supplement: Supplementary Figure 3 — The abundance of several microbial species altered on mouse skin upon topical triple antibiotic application. (A) Lachnospiraceae sp., (B) Elizabethkingia sp., (C) Ruminococcaceae sp., (D) Undibacterium sp., (E) Serratia sp., (F) Cupriavidus sp., (G) Shigella sp., (H) Ruminococcus sp., (I) Methylophilaceae sp. [file Image_3.jpg]

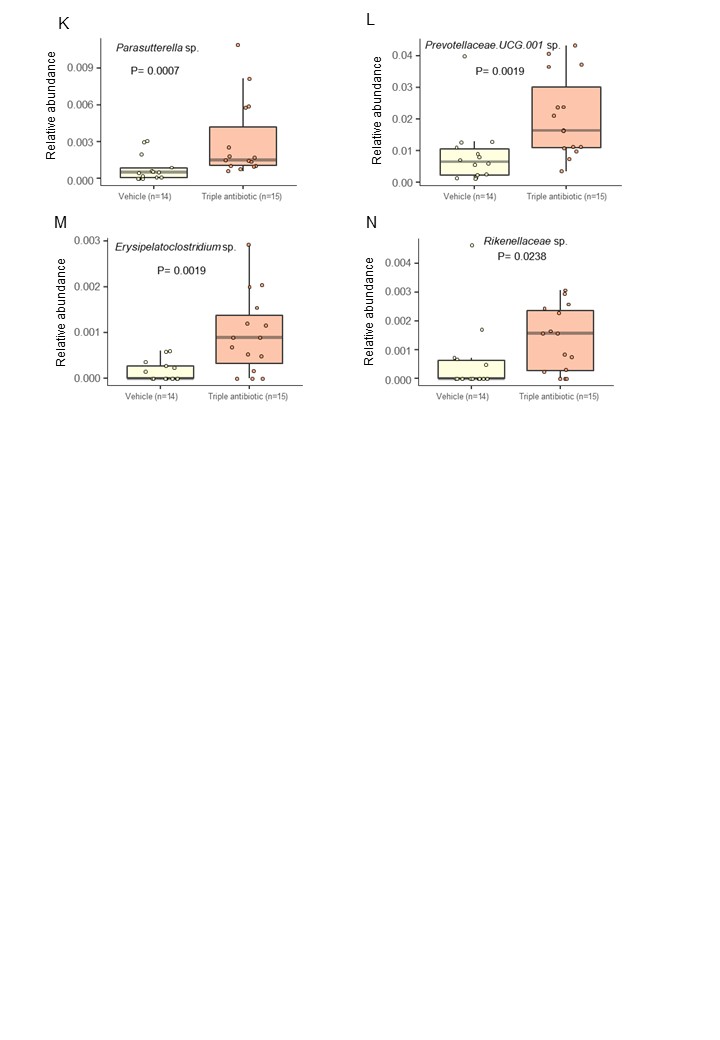

Supplement: Supplementary Figure 4 — Abundance of several commensal microbial species increased significantly on mouse skin upon tropical triple antibiotic application: (A) Alistipes sp., (B) Akkermansia sp., (C) Lachnoclostridium sp., (D) Muribaculum sp., (E) Rhodospirillales sp., (F) Erysipelotrichaceae sp., (G) Parabacteroides sp., (H) Faecalibaculum sp., (I) Marvinbryantia sp., (J) Ruminococcaceae sp., (K). Parasutterella sp., (L) Prevotellaceae sp.,(M) Erysipelatoclostridium sp., (N) Rikenellaceae sp. [file Image_4.jpg]
